# Glutathione-mediated antioxidant response and aerobic metabolism: two crucial factors involved in determining the multi-drug resistance of high-risk neuroblastoma

**DOI:** 10.18632/oncotarget.12209

**Published:** 2016-09-23

**Authors:** Renata Colla, Alberto Izzotti, Chiara De Ciucis, Daniela Fenoglio, Silvia Ravera, Andrea Speciale, Roberta Ricciarelli, Anna Lisa Furfaro, Alessandra Pulliero, Mario Passalacqua, Nicola Traverso, Maria Adelaide Pronzato, Cinzia Domenicotti, Barbara Marengo

**Affiliations:** ^1^ Department of Experimental Medicine, University of Genova, Genova, Italy; ^2^ Department of Health Sciences, University of Genova, Genova, Italy; ^3^ IRCCS AOU San Martino IST Genova, Genova, Italy; ^4^ Center of Excellence for Biomedical Research, Department of Internal Medicine, University of Genova, Genova, Italy; ^5^ Department of Pharmacy, University of Genova, Genova, Italy; ^6^ Giannina Gaslini Institute, Genova, Italy

**Keywords:** neuroblastoma, multi-drug resistance, glutathione, antioxidants, aerobic metabolism

## Abstract

Neuroblastoma, a paediatric malignant tumor, is initially sensitive to etoposide, a drug to which many patients develop chemoresistance. In order to investigate the molecular mechanisms responsible for etoposide chemoresistance, HTLA-230, a human MYCN-amplified neuroblastoma cell line, was chronically treated with etoposide at a concentration that *in vitro* mimics the clinically-used dose. The selected cells (HTLA-Chr) acquire multi-drug resistance (MDR), becoming less sensitive than parental cells to high doses of etoposide or doxorubicin. MDR is due to several mechanisms that together contribute to maintaining non-toxic levels of H_2_O_2_. In fact, HTLA-Chr cells, while having an efficient aerobic metabolism, are also characterized by an up-regulation of catalase activity and higher levels of reduced glutathione (GSH), a thiol antioxidant compound. The combination of such mechanisms contributes to prevent membrane lipoperoxidation and cell death. Treatment of HTLA-Chr cells with L-Buthionine-sulfoximine, an inhibitor of GSH biosynthesis, markedly reduces their tumorigenic potential that is instead enhanced by the exposure to N-Acetylcysteine, able to promote GSH synthesis.

Collectively, these results demonstrate that GSH and GSH-related responses play a crucial role in the acquisition of MDR and suggest that GSH level monitoring is an efficient strategy to early identify the onset of drug resistance and to control the patient's response to therapy.

## INTRODUCTION

Neuroblastoma is a childhood solid tumor originating from progenitor cells of the sympathetic nervous system and accounts for 8-10% of all childhood cancers and 15% of deaths from pediatric cancer [1–3]. It is characterized by a plethora of biological behaviors which range from tumors which regress or differentiate spontaneously into ganglioneuromas to highly aggressive forms which are frequently fatal. High-risk (HR) neuroblastoma is characterized by metastatic disease and/or amplification of the MYCN proto-oncogene that is a biomarker still used today to stratify risk [2–6].

Current treatment for HR patients includes intensive and toxic chemotherapy followed by surgical resection, myeloablation and autologous stem cell rescue, radiation, and intensive immunotherapy [7–9]. Although most HR patients initially respond to chemotherapy, the majority of them relapse and succumb to the therapy-resistant disease [7–9]. Standard chemotherapy for HR patients combines several compounds and among them etoposide is widely used [10–12]. Etoposide has anti-tumor effects both as a single agent and as part of multi-drug regimens, but its side-effects [13, 14] and chemoresistance limit its clinical success [15].

Chemoresistance is a multifactorial phenomenon and the availability of antioxidants is recognized as one of the critical factors able to provide cancer cells with resistance to anticancer therapies.

For this reason, several anticancer drugs produce high levels of reactive oxygen species (ROS), which cause cell death. Unfortunately, cancer cells adapt by up-regulating antioxidant proteins and ROS scavenging systems to keep ROS levels under the cytotoxic limit. Since several studies have reported that chemoresistant phenotypes of cancer cells display high levels of glutathione (GSH) [16], the intracellular oxidative status has been hypothesized to being a marker of drug efficacy in cancer patients [17].

In this context, the aims of the present study are firstly, to select for a MYCN-amplified neuroblastoma cell line resistant to etoposide, in order to investigate the mechanisms of chemoresistance and secondly, to clarify the role of GSH and GSH-related events in the redox homeostasis, potentially responsible for chemoresistance.

## RESULTS

### Chronically-etoposide-treated neuroblastoma cells are less proliferating and tumorigenic than the parental ones and they don't undergo apoptosis after etoposide exposure

In order to select an etoposide-resistant cell line, HTLA-230, MYCN-amplified human neuroblastoma cells, isolated from a stage IV patient, were treated for 6 months with increasing concentrations of etoposide (1 nM-1.25 μM) and then maintained in culture with the same drug at the concentration of 1.25 μM, which mimics the dose commonly used to treat neuroblastoma patients [18].

It should be noted that in all experiments the acutely-etoposide treated HTLA and the chronically-etoposide-treated HTLA (HTLA-Chr) were compared to the untreated parental HTLA, the cells from which both populations derive.

**Figure 1:**
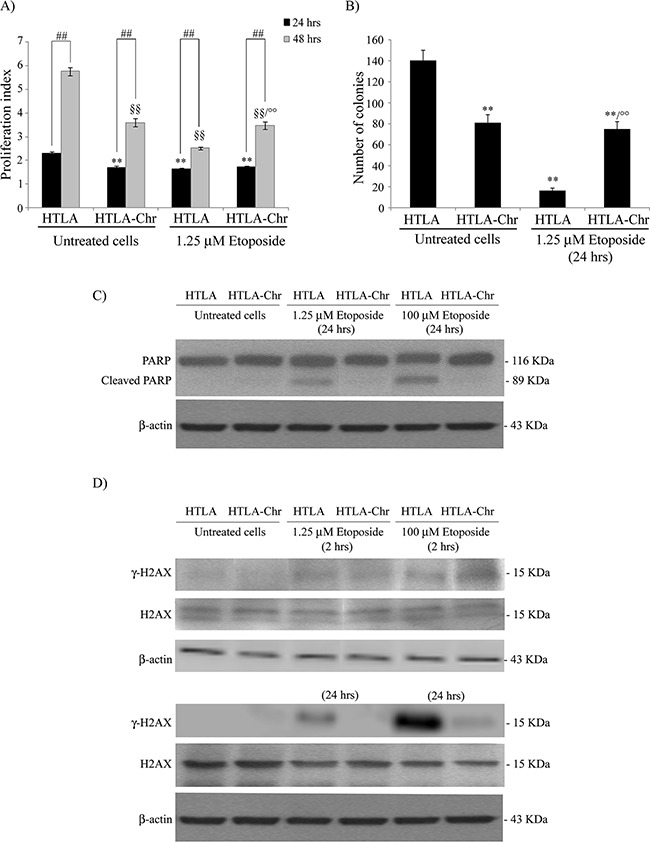
Chronically-etoposide-treated HTLA cells (HTLA-Chr) are less proliferating and tumorigenic than untreated HTLA parental cells and they evade apoptotic death induced by etoposide exposure **A.** Proliferation assay. HTLA parental cells and HTLA-Chr cells were incubated with CFDA-SE and the intensity of cellular CFDA-SE fluorescence was evaluated at 24 hrs and 48 hrs after 1.25 μM etoposide treatment. Results were expressed as proliferation index and are the means ±S.E.M. of three independent experiments. **p<0.01 vs. 24 hrs untreated HTLA cells; °°*p*<0.01 vs. 1.25 μM 48 hrs etoposide-treated HTLA cells; ^##^*p*<0.01 vs. 24 hrs; ^§§^*p*<0.01 vs. 48 hrs untreated HTLA cells. **B.** Clonogenic assay. HTLA parental cells and HTLA-Chr cells were seeded in six-well plates and then incubated with 1.25 μM etoposide for 24 hrs. Subsequently, cells were incubated in fresh medium without the drug for an additional 20 days before staining and counting the colonies. The histogram summarizes quantitative data of the means ± S.E.M. of four independent experiments. **p<0.01 vs. untreated HTLA cells; °°*p*<0.01 vs. 1.25 μM etoposide treated HTLA cells. **C.** Protein levels of PARP in HTLA and HTLA-Chr cells treated for 24 hrs with 1.25 and 100 μM etoposide. Immunoblots shown are representative of three independent experiments. β-Actin is the internal loading control. **D.** Protein levels of γ-H2AX and H2AX in HTLA and HTLA-Chr cells treated for 2 hrs (upper panels) or for 24 hrs (lower panels) with 1.25 and 100 μM etoposide. Immunoblots shown are representative of three independent experiments. β-Actin is the internal loading control.

As shown in Figure 1A, the proliferation rate of HTLA cells, treated with 1.25 μM etoposide for 24 or 48 hrs, was significantly reduced compared to the untreated control cells. Untreated and 24 hr-1.25 μM etoposide-treated HTLA-Chr had a proliferative capacity similar to that of acutely-treated HTLA (Figure 1A). However, after 48 hrs of etoposide treatment, the proliferation index of HTLA-Chr cells was higher than that of HTLA (Figure 1A).

Moreover, the tumorigenic potential of HTLA, indicated by the ability to generate colonies, was reduced by 89% after 24 hrs of etoposide treatment whereas, after the same exposure, the clonogenicity of HTLA-Chr cells was only reduced by 50%, in respect to untreated HTLA cells, remaining 5-fold higher than that of acutely-treated parental cells (Figure 1B). The proliferation rate and the clonogenic potential of HTLA-Chr cells did not change after etoposide exposure (Figures 1A and 1B).

In addition, as shown in Figure 1C, 1.25 μM and 100 μM etoposide treatment for 24 hrs induced apoptosis in HTLA parental cells but not in HTLA-Chr cells, as demonstrated by the PARP cleavage. Interestingly, the expression of γ-H2AX, a marker of DNA double-strand breaks, was induced after 2 hrs of 1.25 and 100 μM etoposide treatment in both cell populations while after 24 hrs it was enhanced in HTLA cells but not detected in HTLA-Chr cells (Figure 1D).

The loss of γ-H2AX expression in etoposide-treated HTLA-Chr cells was accompanied by a 7-9 fold up-regulation of PIM2, RAD54B, DDB1 and FEN1, four genes involved in DNA repair mechanism as shown by the microarray analysis (Table 1).

**Table 1:** Microarray analysis of the DNA repair genes over-expressed in 24 hr etoposide-treated HTLA-Chr cells in comparison with etoposide-treated HTLA parental cells

Gene name	Function	A) Gene expression in 1.25 μM etoposide-treated HTLA-Chr cells (fluorescence Units)	B) Gene expression in 1.25 μM etoposide-treated HTLA cells (fluorescence Units)	Fold increase (column A/B)
PIM2	serine-threonine kinase which mediates DNA damage response *via* ATR [91]	4.16	0.48	8.74 ± 1.1
RAD54B	scaffold for p53 degradation facilitating its ubiquitination [92]	6.56	0.78	8.66 ± 2.0
DDB1	protein involved in the nucleotide excision repair [93]	11.24	1.62	6.94 ± 1.4
FEN1	endonuclease that recognizes and cleaves one nucleotide into the double-stranded DNA junctions [94]	9.860	1.40	7.22 ± 2.3

PIM2, proviral integrations of Moloney virus; DDB1, DNA damage-binding protein 1; FEN1, flap endonuclease 1.

Considering the different response of the two cell populations to etoposide exposure, their ability of internalizing different amounts of etoposide, for the same given dose (1.25 μM), was evaluated. As shown in Supplementary Figure S1, the intracellular (panel A) and the extracellular (panel B) etoposide levels were similar in both cell lines and were constant throughout the 24 hrs of treatment.

### Chronic etoposide treatment induces a multi-drug resistant phenotype

**Figure 2:**
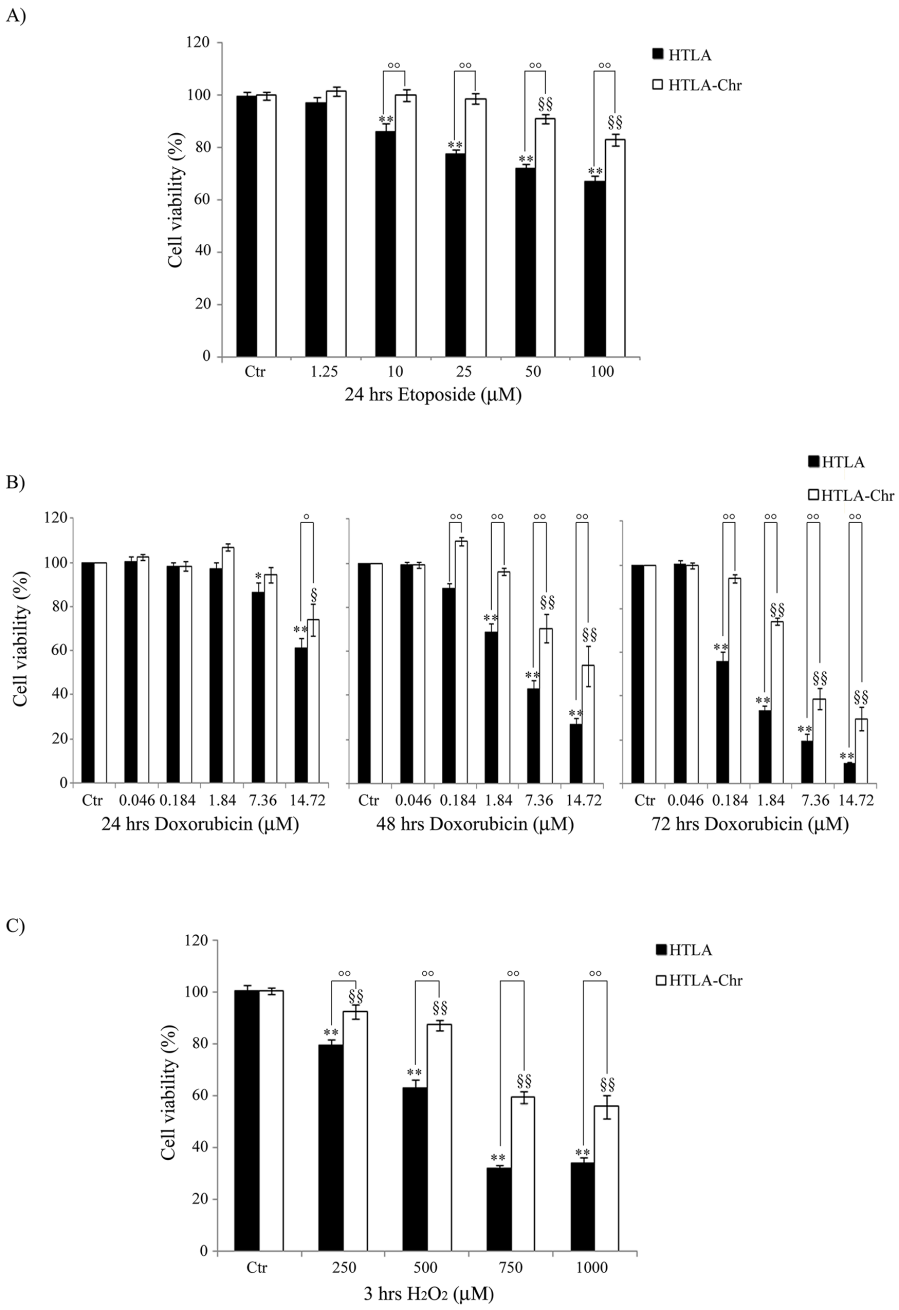
HTLA-Chr cells develop a multi-drug resistant phenotype Cell viability was determined by MTT assays in cells exposed to increasing concentrations of etoposide (1.25–100 μM) for 24 hrs **A.** of doxorubicin (0.046-14.72 μM) for 24, 48 and 72 hrs **B.** and of H_2_O_2_ (250-1000 μM) for 3 hrs **C.** Histograms summarize quantitative data of the means ± S.E.M. of four independent experiments. *p<0.05 vs. untreated HTLA cells (Ctr); **p<0.01 vs. untreated HTLA cells (Ctr); °*p*<0.05 vs. treated HTLA cells; °°*p*<0.01 vs. treated HTLA cells; ^§^*p*<0.05 vs. untreated HTLA-Chr cells (Ctr); ^§§^*p*<0.01 vs. untreated HTLA-Chr cells (Ctr).

To evaluate the degree of resistance to etoposide, HTLA and HTLA-Chr cells were exposed to increasing concentrations (1.25 μM-100 μM) of the drug for 24 hrs. As shown in Figure 2A, etoposide was cytotoxic for HTLA cells in a concentration-dependent manner. In fact, 10 μM etoposide decreased the viability of HTLA cells by 14% and the highest dose (100 μM) of the drug led to 35% of cell death. In HTLA-Chr, the cytotoxic effect was recorded only at the doses of 50 and 100 μM, with a 9% and 17% reduction in cell viability, respectively (Figure 2A).

To investigate the hypothesis of multi-drug resistance, HTLA parental and HTLA-Chr cells were exposed to doxorubicin, another chemotherapeutic drug commonly used in the therapy of neuroblastoma [19]. Figure 2B shows that 0.184 μM doxorubicin reduced the viability of HTLA parental cells by 44% only after 72 hrs whilst did not affect the viability of HTLA-Chr cells. The highest doxorubicin dose (14.72 μM) was cytotoxic for both cell populations already after 24 hrs inducing 40% and 25% cell death in HTLA parental and HTLA-Chr cells, respectively. Cell viability was further decreased after 48 hrs of treatment, and reached 90% and 70% of reduction in HTLA parental and HTLA-Chr cells, respectively, after 72 hrs (Figure 2B).

H_2_O_2_ exposure (3 hrs) also induced a concentration-dependent reduction of cell viability in both cell populations (Figure 2C) and yet, cytotoxicity was higher in HTLA parental cells where 1000 μM H_2_O_2_ reduced the viability by 66% compared to 44% of HTLA-Chr cells.

In order to confirm the MDR phenotype of HTLA-Chr cells, a microarray analysis of genes involved in MDR system was performed. Among these genes, ARNT, Ezrin, ABCB6 and BCRP1 (also known as ABCG2), were found to be over-expressed (about 5-15-fold) in 1.25 μM etoposide-treated HTLA-Chr cells in respect to etoposide-treated parental cells (Table 2). These results were confirmed by a real time PCR analysis (Table 2).

**Table 2:** Microarray analysis of the MDR genes over-expressed in 24 hr etoposide-treated HTLA-Chr cells in comparison with etoposide-treated HTLA parental cells

Gene name	A) Gene expression in 1.25 μM etoposide-treated HTLA-Chr cells (fluorescence Units)	B) Gene expression in 1.25 μM etoposide-treated HTLA cells (fluorescence Units)	Fold increase (column A/B)	Fold increase (Real Time RT-PCR)
ARNT	8.004	0.527	15.2 ± 1.7	3.47 ± 1.0
Ezrin	6.469	0.687	9.4 ± 0.9	2.15 ± 0.6
ABCB6	5.419	0.859	6.3 ± 0.5	4.08 ± 1.5
BCRP1	4.746	0.899	5.15 ± 0.6	3.34 ± 0.7

ARNT, Aryl Hydrocarbon receptor Nuclear Translocator; BCRP1/ABCG2, Breast Cancer Resistance Protein 1; ABCB6, mitochondrial ATP binding cassette transporter 6.

### Exposure to etoposide or doxorubicin does not stimulate the ability of HTLA-Chr cells to produce H_2_O_2_

**Figure 3:**
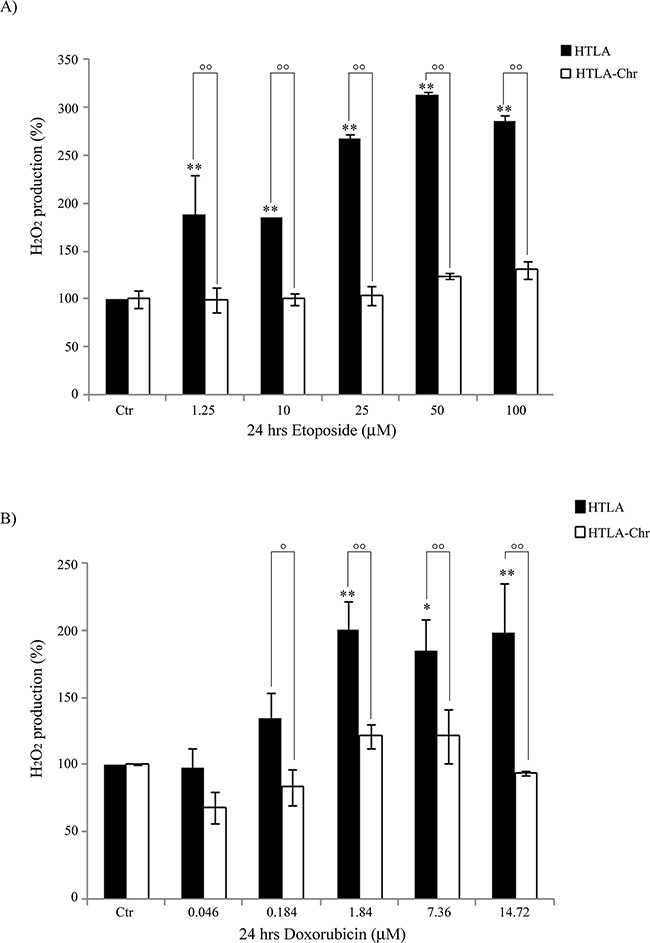
HTLA-Chr cells do not change H_2_O_2_ production after treatment with etoposide or doxorubicin H_2_O_2_ production was analyzed in HTLA and in HTLA-Chr cells incubated for 24 hrs with increasing concentrations of etoposide (1.25–100 μM) **A.** or doxorubicin (0.046-14.72 μM) **B.** Histograms summarize quantitative data of the means ± S.E.M. of three independent experiments. *p<0.05 vs. untreated HTLA cells (Ctr); **p<0.01 vs. untreated HTLA cells (Ctr); °*p*<0.05 vs. treated HTLA cells; °°*p*<0.01 vs. treated HTLA cells.

As shown in Figure 3A, 24 hr etoposide treatment of HTLA parental cells increased the production of H_2_O_2_in a concentration-dependent manner. In particular, compared to untreated HTLA cells, the intracellular peroxide concentration was increased by 88% at the dose of 1.25 μM and further increased by 2-fold at the doses of 50 and 100 μM. Interestingly, the same treatments did not stimulate the production of H_2_O_2_ in HTLA-Chr cells (Figure 3A). A similar result was observed when treating the two cell populations with increasing concentrations of doxorubicin (Figure 3B).

**Figure 4:**
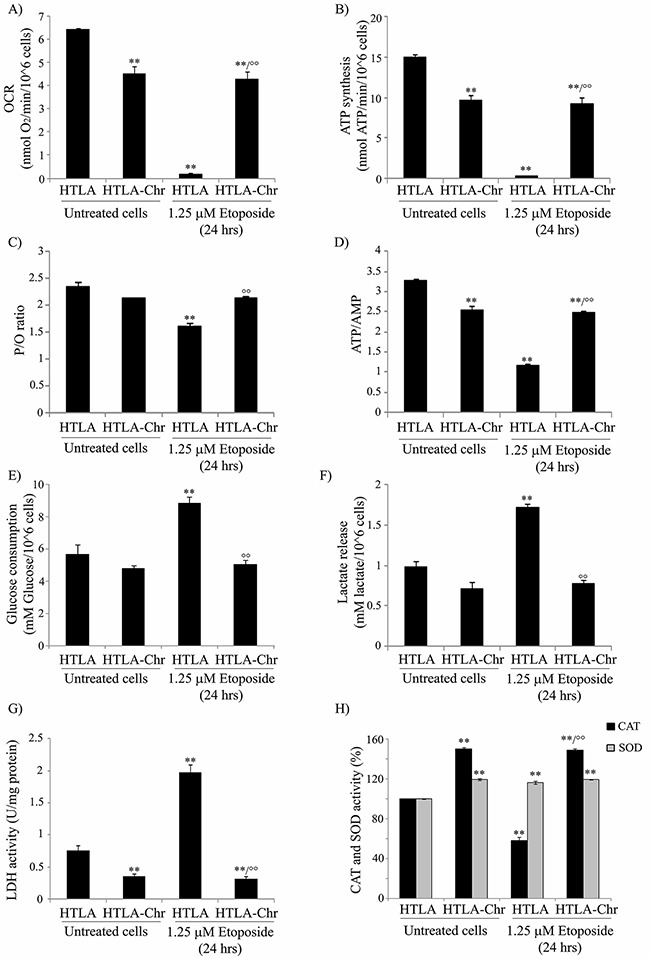
HTLA-Chr cells have a major oxygen consumption, an increased oxidative phosphorylation and an up-regulation of catalase activity **A.** The oxygen consumption rate (OCR) was evaluated in untreated and in 1.25 μM etoposide-treated (24 hrs) HTLA and HTLA-Chr cells. Results were reported as nmol O_2_/min/10^6^ cells. Histogram summarizes quantitative data of means ± S.E.M. of three independent experiments. **p<0.01 vs. untreated HTLA cells; °°*p*<0.01 vs. 1.25 μM etoposide-treated HTLA cells. **B.** ATP synthesis was measured in untreated and in 1.25 μM etoposide-treated (24 hrs) HTLA and HTLA-Chr cells. Results were reported as nmol ATP/min/10^6^ cells. Histogram summarizes quantitative data of means ± S.E.M. of three independent experiments. **p<0.01 vs. untreated HTLA cells; °°*p*<0.01 vs. 1.25 μM etoposide-treated HTLA cells. **C.** P/O is expressed as the ratio of nmol of ATP, measured by luminometry in 1 minute over nmol of O_2_ consumed in 1 minute in the presence of the substrate + ADP. **p<0.01 vs. untreated HTLA cells; °°*p*<0.01 vs. 1.25 μM etoposide-treated HTLA cells. **D.** ATP and AMP were evaluated in untreated and in 1.25 μM etoposide-treated (24 hrs) HTLA and HTLA-Chr cells. Histogram summarizes quantitative data of means ± S.E.M. of three independent experiments. **p<0.01 vs. untreated HTLA cells; °°*p*<0.01 vs. 1.25 μM etoposide-treated HTLA cells. **E.** Glucose consumption was evaluated in untreated and in 1.25 μM etoposide-treated (24 hrs) HTLA and HTLA-Chr cells. Results were reported as mM glucose/10^6^ cells. Histogram summarizes quantitative data of means ± S.E.M. of three independent experiments. **p<0.01 vs. untreated HTLA cells; °°*p*<0.01 vs. 1.25 μM etoposide-treated HTLA cells. **F.** Extracellular lactate concentration was evaluated in untreated and in 1.25 μM etoposide-treated (24 hrs) HTLA and HTLA-Chr cells. Results were reported as mM lactate/10^6^ cells. Histogram summarizes quantitative data of means ± S.E.M. of three independent experiments. **p<0.01 vs. untreated HTLA cells; °°*p*<0.01 vs. 1.25 μM etoposide-treated HTLA cells. **G.** Lactate dehydrogenase activity (LDH) was evaluated in untreated and in 1.25 μM etoposide-treated (24 hrs) HTLA and HTLA-Chr cells. Results were reported as U/mg (lactate μmol/min/mg of total protein). Histogram summarizes quantitative data of means ± S.E.M. of three independent experiments. **p<0.01 vs. untreated HTLA cells; °°*p*<0.01 vs. 1.25 μM etoposide-treated HTLA cells. **H.** Catalase and SOD activities in untreated and in 1.25 μM etoposide-treated (24 hrs) HTLA and HTLA-Chr cells. Histograms summarize quantitative data of means, normalized to the activity of enzymes in untreated cells (100%) ± S.E.M. of three independent experiments. **p<0.01 vs. untreated HTLA cells; °°*p*<0.01 vs. 1.25 μM etoposide-treated HTLA cells.

To investigate the role of cell metabolism in H_2_O_2_ generation, the oxygen consumption rate (OCR) and ATP synthesis were measured. We found that in etoposide-treated HTLA-Chr cells, such parameters were decreased by 34% and 44%, respectively, in comparison to untreated HTLA parental cells, but they were almost abolished in HTLA parental cells acutely-treated with etoposide (Figures 4A and 4B). Moreover, the OCR and ATP synthesis were similar in etoposide-treated HTLA-Chr and in untreated HTLA-Chr cells (Figures 4A and 4B).

To verify the efficiency of the oxidative phosphorylation, the P/O value was evaluated (Figure 4C). In untreated and etoposide-treated HTLA-Chr cells, the P/O ratio was 2.3±0.2 which was comparable to the physiological level (2.5) reported by Hinkle [20]. By contrast, in acutely-treated HTLA parental cells, the P/O value was lower (1.7±0.15) suggesting that a part of the oxygen consumption could be attributed to the production of H_2_O_2_.

Moreover, in order to verify the energetic cellular status, the ATP/AMP ratio was evaluated. As shown in Figure 4D, the ATP/AMP ratio was reduced by 25% in etoposide-treated HTLA-Chr cells and by 67% in acutely-treated HTLA parental cells, confirming that in the latter case, the oxidative phosphorylation was not completely efficient. Also in this case, the ATP/AMP ratio in etoposide-treated HTLA-Chr cells was comparable to that of untreated HTLA-Chr (Figure 4D).

In addition, the rate of glucose consumption, lactate formation, as well as the activity of lactate dehydrogenase (LDH), increased by 56%, 77% and 164%, respectively in acutely-treated HTLA parental cells (Figure 4E, 4F and 4G), suggesting that the impairment of oxidative phosphorylation could be partially compensated by an increment in the anaerobic glycolytic rate.

Since the amount of peroxides is a balance between the mitochondrial ROS formation and ROS detoxification, the status of the main antioxidant enzymes was investigated.

Due to the fact that the gene expression profile analysis demonstrating that SOD1 and catalase expression had been increased by 67% and 71%, respectively in etoposide-treated HTLA-Chr cells compared to acutely-treated parental ones, both enzymatic activities were measured. While total SOD activity was increased by 17% in both etoposide-treated parental and HTLA-Chr cells (Figure 4H), catalase activity was reduced by 42% in acutely-treated HTLA and stimulated by 49% in HTLA-Chr cells exposed to etoposide in respect to HTLA parental ones (Figure 4H). Moreover, SOD and catalase activities in etoposide-treated HTLA-Chr cells were comparable to those of untreated HTLA-Chr (Figure 4H).

### HTLA-Chr cells detoxify H_2_O_2_ and prevent lipid peroxidation by enhancing GSH levels and up-regulating GST activities

**Figure 5:**
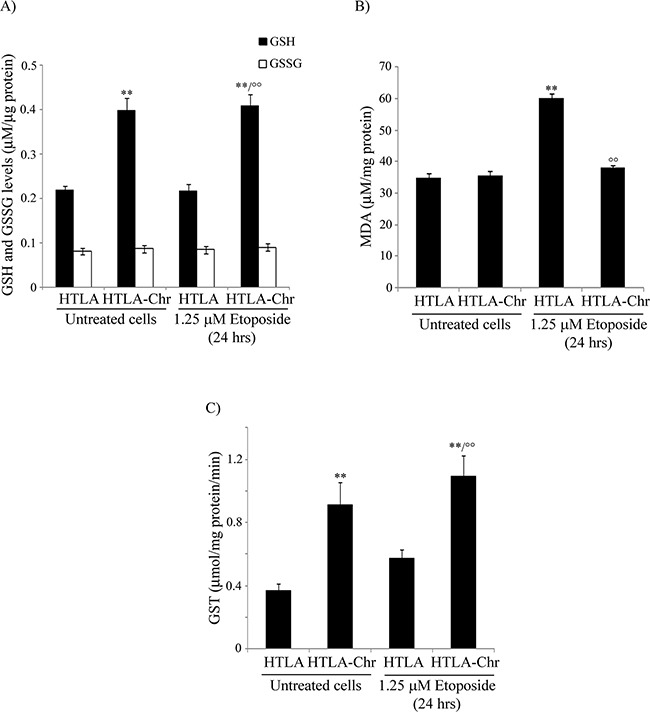
HTLA-Chr cells are characterized by higher GSH levels, a lower amount of a lipid peroxidation marker and up-regulation of GST activity **A.** Reduced and oxidized glutathione (GSH and GSSG) levels were analyzed in untreated and in 1.25 μM etoposide-treated (24 hrs) HTLA and HTLA-Chr cells. Results were reported as μM/μg protein. Histogram summarizes quantitative data of means ± S.E.M. of six independent experiments. **p<0.01 vs. untreated HTLA cells; °°*p*<0.01 vs. 1.25 μM etoposide-treated HTLA cells. **B.** MDA production was evaluated in untreated and in 1.25 μM etoposide-treated (24 hrs) HTLA and HTLA-Chr cells. Results were reported as μM/mg protein. Histogram summarizes quantitative data of means ± S.E.M. of three independent experiments. **p<0.01 vs. untreated HTLA cells; °°*p*<0.01 vs. 1.25 μM etoposide-treated HTLA cells. **C.** GST analysis in untreated and in 1.25 μM etoposide-treated (24 hrs) HTLA and HTLA-Chr cells. Results were reported as μmol/mg prot/min. of reaction product taking into account the conjugated 5-thio-2-nitrobenzoic acid molar extinction coefficient corresponding to 9.6 mol^−1^cm^−1^. Results are the mean ± S.E.M. of six independent analyses. **p<0.01 vs. untreated HTLA cells; °°*p*<0.01 vs. 1.25 μM etoposide-treated HTLA cells.

In untreated and in etoposide-treated HTLA-Chr cells, GSH levels were increased by 85% in respect to both untreated and acutely-treated parental cells while no difference in GSSG levels was observed (Figure 5A).

The analysis of γ-glutamyl-cysteinyl ligase (GCL) expression, a crucial enzyme involved in GSH biosynthesis, showed that the mRNA of catalytic subunit (GCLC) was up-regulated by 20% in untreated and etoposide-treated HTLA-Chr cells compared to HTLA parental ones (Supplementary Figure S2). Conversely, the protein level of GCLC was down-regulated by 85% in untreated and etoposide-treated HTLA-Chr cells in comparison to HTLA parental ones (Supplementary Figure S2). However, mRNA and protein levels of GCLM (modulatory subunit) were similar in parental HTLA and HTLA-Chr cells (data not shown).

In conformity with the trend of GSH levels, the production of MDA, a known lipid peroxidation marker, was increased by 72% in acutely-treated parental cells in respect to untreated ones while it did not change in untreated and etoposide-treated HTLA-Chr cells which maintained the levels found in parental cells (Figure 5B).

Since GSH amount was different in the cell populations analyzed, the expression and the activity of glutathione S-transferase (GST), a phase II detoxification enzyme that catalyzes the conjugation of GSH with exogenous and endogenous electrophilic substrates, was investigated. In fact, microarray analysis showed that the GST gene in etoposide-treated HTLA-Chr cells was over-expressed by 2.2-fold in respect to acutely-treated parental cells and in accordance with this data, GST activity was increased 2-fold (Figure 5C).

### GSH depletion by BSO increases H_2_O_2_ levels and markedly reduces the tumorigenic potential of HTLA-Chr cells

In order to test the role of GSH in the acquisition of drug resistance, both HTLA parental and HTLA-Chr cells were pre-treated for 1 hr with 1 mM L-buthionine-sulfoximine (BSO), a GSH depleting agent and then exposed to etoposide for 24 hrs.

**Figure 6:**
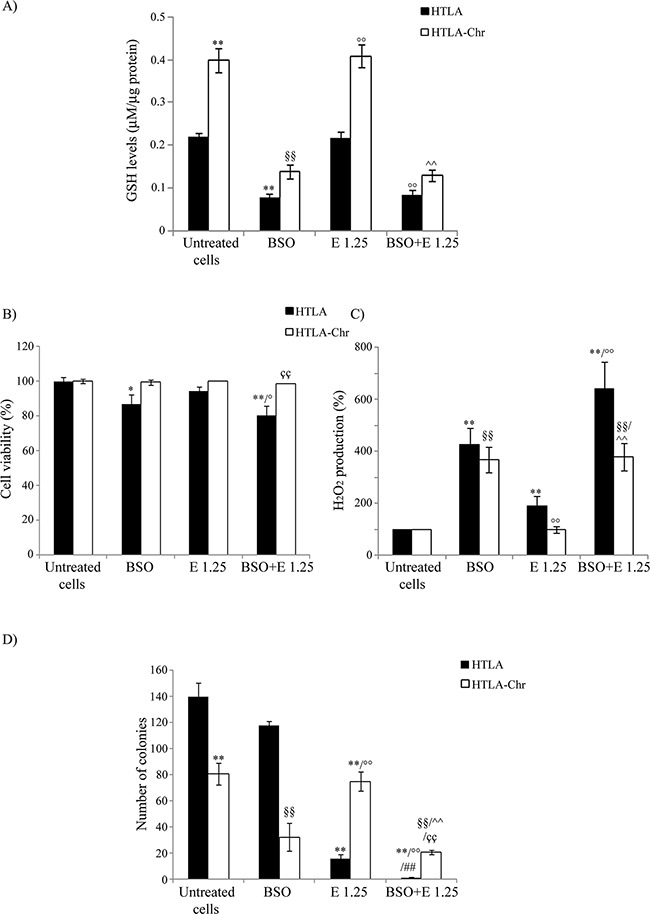
BSO treatment induces GSH depletion, increases H_2_O_2_ production and markedly reduces the tumorigenic potential of etoposide-resistant cells **A.** GSH levels were analyzed in HTLA and HTLA-Chr cell treated with 1 mM BSO or pre-treated (1 hr) with 1 mM BSO and then exposed (24 hrs) to 1.25 μM etoposide. Results were reported as μM/μg protein. Histogram summarizes quantitative data of the means ± S.E.M. of three independent experiments. **p<0.01 vs. untreated HTLA cells; ^§§^*p*<0.01 vs. untreated HTLA-Chr cells; °°*p*<0.01 vs. 1.25 μM etoposide-treated HTLA cells; ^^*p*<0.01 vs. 1.25 μM etoposide-treated HTLA-Chr cells. **B.** Cell viability was determined by MTT assays in HTLA and HTLA-Chr cells treated with 1 mM BSO or pre-treated (1 hr) with 1 mM BSO and then exposed (24 hrs) to 1.25 μM etoposide. Histogram summarizes quantitative data of the means ± S.E.M. of four independent experiments. *p<0.05 vs. untreated HTLA cells; **p<0.01 vs. untreated HTLA cells; °*p*<0.05 vs. 1.25 μM etoposide-treated HTLA cells; ^çç^*p*<0.01 vs. HTLA cells pre-treated with 1 mM BSO and then exposed to 1.25 μM etoposide. **C.** H_2_O_2_ production was analyzed in HTLA and HTLA-Chr cells treated with 1 mM BSO or pre-treated (1 hr) with 1 mM BSO and then exposed (24 hrs) to 1.25 μM etoposide. Histogram summarizes quantitative data of the means ± S.E.M. of four independent experiments. ** p<0.01 vs. untreated HTLA cells; °° p<0.01 vs. etoposide-treated HTLA cells; §§p<0.01 vs. untreated HTLA-Chr cells; ^^p<0.01 vs. 1.25 μM etoposide-treated HTLA-Chr cells. **D.** Clonogenic assay was carried out in HTLA and HTLA-Chr cells treated with 1mM BSO or pre-treated (1 hr) with 1 mM BSO and then exposed (24 hrs) to 1.25 μM etoposide. Subsequently, cells were incubated in fresh medium without the drug for an additional 20 days before staining and counting the colonies. The histogram summarizes quantitative data of the means ± S.E.M. of three independent experiments. **p<0.01 vs. untreated HTLA cells; °°*p*<0.01 vs. etoposide-treated HTLA cells; ^§§^*p*<0.01 vs. untreated HTLA-Chr cells; ^^*p*<0.01 vs. 1.25 μM etoposide-treated HTLA-Chr cells; ^##^*p*<0.01 vs. 1mM BSO-treated HTLA cells; ^çç^*p*<0.01 vs. HTLA cells pre-treated with 1 mM BSO and then exposed to 1.25 μM etoposide.

As shown in Figure 6A, exposure to 1 mM BSO alone reduced the GSH levels of parental cells by 65%. Moreover, BSO reduced the GSH content of parental and HTLA-Chr cells, treated for 24 hrs with 1.25 μM etoposide, by 63% and 70%, respectively (Figure 6A). BSO-induced changes in GSH levels were comparable in etoposide-treated HTLA-Chr cells and in untreated HTLA-Chr (Figure 6A).

In observing Figure 6B, BSO treatment decreased the parental cell viability by 14% compared to control and sensitized them to etoposide. Inversely, BSO did not modify the sensitivity of HTLA-Chr cells to the cytotoxic drug (Figure 6B).

As shown in Figure 6C, parental cells, treated with BSO, increased H_2_O_2_ production by 3.2-fold compared to untreated parental ones. Moreover, in the same cells, BSO-etoposide co-treatment increased peroxide levels by 2.4-fold in respect to the etoposide alone (Figure 6C). Similarly, in HTLA-Chr cells, BSO co-treatment stimulated H_2_O_2_ production by 2.6-fold compared to etoposide-treated HTLA-Chr cells (Figure 6C). BSO-mediated peroxide over-production was similar in etoposide-treated HTLA-Chr cells and in untreated HTLA-Chr (Figure 6C).

BSO treatment*per se* did not affect the clonogenic potential of HTLA parental cells, but almost abolished the clonogenicity of the same cells acutely-exposed to etoposide while reduced the clonogenicity of etoposide-treated HTLA-Chr cells by 73% (Figure 6D). The reduction of clonogenic potential by BSO was found to be comparable in etoposide-treated HTLA-Chr cells and in untreated ones (Figure 6D).

### Increasing GSH by NAC prevents H_2_O_2_ increase and markedly enhances the tumorigenic potential of HTLA-Chr cells

In order to further investigate the role of GSH in drug resistance, both cell populations were pre-treated for 1 hr with 2 mM N-Acetylcysteine (NAC), an aminothiol and synthetic precursor of intracellular cysteine and then exposed to etoposide for 24 hrs.

**Figure 7:**
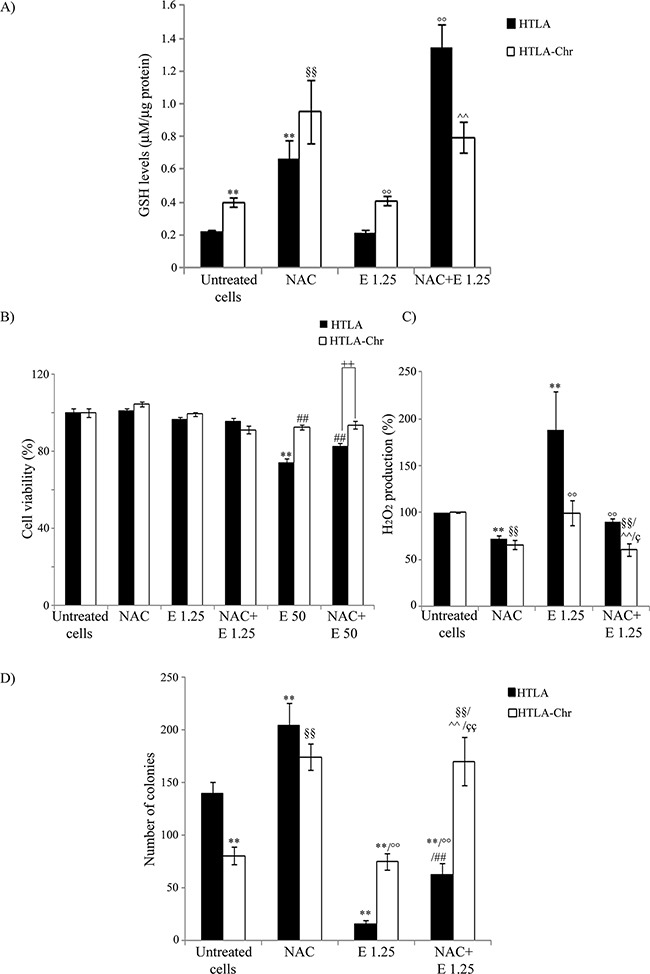
NAC treatment enhances GSH levels, decreases H_2_O_2_ production and markedly promotes the tumorigenic potential of neuroblastoma cells **A.** GSH levels were analyzed in HTLA and HTLA-Chr cells treated with 2 mM NAC or pre-treated (1 hr) with 2 mM NAC and then exposed (24 hrs) to 1.25 μM etoposide. Histogram summarizes quantitative data of the means ± S.E.M. of three independent experiments. **p<0.01 vs. untreated HTLA cells; ^§§^*p*<0.01 vs. untreated HTLA-Chr cells; °°*p*<0.01 vs. 1.25 μM etoposide-treated HTLA cells, ^^*p*<0.01 vs. 1.25 μM etoposide-treated HTLA-Chr cells. **B.** Cell viability was determined by MTT assays in HTLA and HTLA-Chr cells treated with 2 mM NAC or pre-treated (1 hr) with 2 mM NAC and then exposed (24 hrs) to 1.25 μM and 50 μM etoposide. Histogram summarizes quantitative data of the means ± S.E.M. of four independent experiments. **p<0.01 vs. untreated HTLA cells; ^##^*p*<0.01 vs. 50 μM etoposide-treated HTLA cells; ^++^*p*<0.01 vs. HTLA cells. **C.** H_2_O_2_ production was analyzed in HTLA and HTLA-Chr cells treated with 2 mM NAC or pre-treated (1 hr) with 2 mM NAC and then exposed (24 hrs) to 1.25 μM etoposide. Histogram summarizes quantitative data of means ± S.E.M. of four independent experiments. **p<0.01 vs. untreated HTLA cells; °°*p*<0.01 vs. etoposide-treated HTLA cells; ^§§^*p*<0.01 vs. untreated HTLA-Chr cells; ^^*p*<0.01 vs. 1.25 μM etoposide-treated HTLA-Chr cells; ^ç^*p*<0.05 vs. HTLA cells pre-treated with NAC and then exposed to 1.25 μM etoposide. **D.** Clonogenic assay was carried out in HTLA and HTLA-Chr cells treated with 2 mM NAC or pre-treated (1 hr) with 2 mM NAC and then exposed (24 hrs) to 1.25 μM etoposide. Subsequently, cells were incubated in fresh medium without the drug for an additional 20 days before staining and counting the colonies. The histogram summarizes quantitative data of the means ± S.E.M. of three independent experiments. **p<0.01 vs. untreated HTLA cells; °°*p*<0.01 vs. etoposide-treated HTLA cells; ^§§^*p*<0.01 vs. untreated HTLA-Chr cells; ^^*p*<0.01 vs. 1.25 μM etoposide-treated HTLA-Chr cells; ^##^*p*<0.01 vs. NAC-treated HTLA cells; ^çç^*p*<0.01 vs. HTLA cells pre-treated with NAC and then exposed to 1.25 μM etoposide.

As shown in Figure 7A, NAC increased the GSH levels of parental cells by 200%. Moreover, this rate of increase reached 500% when the cells having been pre-treated with NAC were exposed for 24 hrs to etoposide. However, a more modest effect was observed in etoposide-treated HTLA-Chr cells where NAC co-treatment increased GSH levels by 100% (Figure 7A). NAC partially protected parental cells from the cytotoxicity induced by 50 μM etoposide but it did not modify the viability of HTLA-Chr cells (Figure 7B).

In addition, NAC reduced the peroxide production in HTLA and HTLA-Chr cells by 28% and, in the acutely-etoposide-treated HTLA cells, by 55% (Figure 7C). A similar, but less marked effect was observed in etoposide-treated HTLA-Chr cells (Figure 7C).

NAC alone was able to increase the clonogenic potential of parental cells by 45% and of HTLA-Chr cells by 110% (Figure 7D) and this effect was more evident in cells co-treated with etoposide. In particular, NAC-etoposide co-treatment increased the clonogenicity of both acutely and chronically-etoposide-treated HTLA cells by 300% and 126%, respectively (Figure 7D).

## DISCUSSION

Neuroblastoma, the second most common childhood solid malignant tumor, is clinically characterized by a wide heterogeneity. To date, several drugs are available and among them etoposide, an inhibitor of topoisomerase II, is the standard clinically-used chemotherapeutic compound. Initially, patients are sensitive to this drug but unfortunately about 15-20% of them develop chemoresistance [13, 14, 21].

In order to investigate the molecular mechanisms of chemoresistance, we chronically exposed a MYCN-amplified human neuroblastoma cell line, isolated from a stage IV patient, to etoposide [22]. By using a concentration of etoposide that mimics the clinically-used dose [18], we selected a cell population which might represent a good model for studying chemoresistance *in vitro*. In a previous study, Urbani et al. selected MYCN non-amplified neuroblastoma cells resistant to etoposide and they identified by proteomic analysis some potential markers of drug resistance such as Hsp27, beta-galactoside soluble lectin binding protein, vimentin, heterogeneous nuclear ribonucleoprotein K and peroxiredoxin [23]. The microarray analysis of gene profile in HTLA-Chr cells shows that the expression of these markers is not modified. Therefore, we believe that the discrepancy between our results and those of Urbani et al. might be due to the fact that HTLA-Chr cells are MYCN-amplified, while SH-SY-5Y cells, the neuroblastoma line used by Urbani, are MYCN-unamplified. In support of the influence exerted by MYCN on the induction of a different gene profile, an inverse correlation between MYCN amplification and Hsp27 has been described [24] and this data might well explain the overexpression of this protein in SH-SY-5Y-resistant cells but not in HTLA-Chr cells. Moreover, it has been found that SH-SY-5Y neuroblastoma cells do not express vimentin [25] and that the selection with etoposide is able to stimulate vimentin which is basally expressed in HTLA cells [26]. Although useful, Urbani's model was not representative of the more aggressive and therapy-refractory forms of neuroblastoma, characterized by the amplification of the MYCN proto-oncogene [27–30].

Herein, we demonstrate that HTLA-Chr cells are less proliferating than parental cells. This is in line with a previous report showing that anticancer therapy, administered for a prolonged period, leads cancer cells into a slow proliferating state, rendering them less sensitive to chemotherapy-induced effects [31].

In addition, HTLA-Chr cells are more tumorigenic than parental cells acutely treated with etoposide, and become resistant to the pro-apoptotic effect of etoposide so demonstrating that the chronic treatment with the drug is able to select a more malignant cell population. Although etoposide in all cell populations is internalized in similar amounts, only HTLA-Chr cells survive under etoposide treatment thanks to their ability to efficiently repair DNA damage and so evading the apoptotic death. In fact, the microarray analysis shows that genes such as PIM2, RAD54B, DDB1 and FEN1 involved in DNA repair, are overexpressed in HTLA-Chr cells which become resistant to the genotoxic effect of etoposide. A similar over-expression of DNA repair genes has been observed also in tumor cells that were resistant to cisplatin treatment [32]. Therefore, our findings are consistent with other studies which report that an efficient ability to repair DNA damage results in chemoresistance [33] while a defective DNA repair capacity is responsible for the cytotoxic effect of drugs [34].

Based on the fact that chemoresistance often transforms into multi-drug resistance (MDR), which characterizes highly malignant neuroblastoma [35, 36], HTLA-Chr and parental cells were exposed to high doses of etoposide or doxorubicin using concentrations equal to, or higher than, those used to treat patients [18, 37].

Our results clearly show that HTLA-Chr cells acquire the MDR phenotype in that they are less sensitive than parental cells to high doses of either etoposide or doxorubicin. Etoposide and doxorubicin-induced cell death is, at least in part, mediated by oxidative stress [15, 38–40]. Interestingly, we have demonstrated that HTLA-Chr cells are less sensitive to pro-oxidant treatments (i.e. etoposide or doxorubicin or even H_2_O_2_) than the parental cells. Moreover, the analysis of the impact by chemotherapeutic drugs on the cellular oxidative status revealed that HTLA-Chr cells maintain basal levels of H_2_O_2_ after treatment with etoposide or doxorubicin, whereas the parental cells markedly increase their H_2_O_2_ production. Accordingly, recent findings demonstrate that, although most of the anticancer drugs kill cancer cells by inducing oxidative stress, prolonged treatments lead to a reduced oxidative stress as a consequence of therapy resistance [41, 42]. It was also demonstrated that the acquisition of chemoresistance in gliomas is associated with decreased ROS production and increased mitochondrial coupling [43]. In our study, HTLA-Chr cells show a high respiratory rate in terms of oxygen consumption and P/O ratio which could explain the lower peroxide production. In line with this hypothesis, the high efficiency of aerobic metabolism in HTLA-Chr cells is paralleled by a lower anaerobic glycolytic rate as measured in terms of lactate formation, LDH activity and glucose consumption.

Another strategy developed by cancer cells in order to maintain peroxides within non-toxic levels is that of up-regulating scavenging enzymes, such as SOD and catalase, also involved in drug resistance [44–47].

Our results, showing that chronic etoposide treatment strongly increases the activity of catalase, which degrades peroxides,contribute to explaining the reduced presence of H_2_O_2_ in HTLA-Chr cells. These findings are also in line with a study demonstrating that chemoresistance of lymphoma cells results from the concomitant increase of SOD, which generates H_2_O_2_, and catalase activity [48].

To further investigate the antioxidant systems of our cellular model, we analyzed the role of glutathione (GSH), an ubiquitous thiol involved in detoxification, redox regulation and cellular signaling [16]. In accordance with the fact that GSH conjugation may be responsible for drug-resistant phenotypes and non-responsiveness of brain tumors to alkylating agents [49], our results demonstrate that HTLA-Chr cells are characterized by higher levels of GSH, compared to untreated and acutely-etoposide treated parental cells. This is in accordance with other findings stating that high levels of GSH in tumors are linked to the development and expression of MDR [50] and elevated levels of GSH are directly correlated with resistance to camptothecin (CPA) and 4-hydroperoxy-CPA (4-HC) in a panel of medulloblastoma cell lines [51].

In our context, however, the high amount of GSH in HTLA-Chr cells is not directly related to an increased expression of g-glutamyl-cysteinyl ligase (GCL), a rate-limiting enzyme in GSH biosynthesis [52]. In fact, our findings, also in line with a recent study [53], demonstrate that the increase in GCLC mRNA is followed by a decrease in GCLC protein level, whereas GCLM mRNA and protein level have a similar trend of expression.

Some authors suggest that GSH-mediated drug resistance might be the consequence of changes in GSH-related enzyme activities [51, 54, 55]. In detail, it has been demonstrated that cancer cells resistant to 1,3-bis(2-chloroethiyl)-1-nitrosourea (BCNU) [54, 55] are characterized by high levels of GSH and a major activity of GST enzyme which promotes GSH conjugation with the drug contributing to its inactivation [54]. In addition, chemoterapy-resistant orthotopic xenografts of nonsmall cell lung carcinoma were seen to express high GST levels [56] and in accordance with these observations, our own results show that HTLA-Chr cells have a higher GST level/activity than the parental cells.

Therefore, our data leads us to hypothesizing that the MDR of HTLA-Chr cells is dependent on GSH, since it is responsible for the increase in GST activity which is potentially involved in the acquisition of the MDR phenotype.

Among MDR genes analyzed, we have taken into consideration the genes related to GSH and over-expressed in HTLA-Chr cells. In particular, ARNT (Aryl Hydrocarbon Receptor Nuclear Translocator) is a nuclear receptor which has been demonstrated to enhance antioxidant response and to confer drug resistance in leukemia cells [57]. ARNT is also involved in the up-regulation of BCRP1/ABCG2 (Breast Cancer Resistance Protein 1) [58, 59], a drug transporter which can also mediate GSH transport [60]. Multidrug transporters include P-glycoprotein (Pgp), a protein efflux pump belonging to the ATP-binding cassette (ABC) superfamily, which binds to Ezrin, a cytoskeletal protein correlated with the metastatic phenotype and multidrug resistance of lymphoid cells [61]. Furthermore, the acquisition of chemoresistance in HTLA-Chr cells is associated with the overexpression of ABCB6 (ATP binding cassette transporter 6), a mitochondrial drug transporter which is also correlated with the multidrug resistance in several cancer cells [62, 63].

On the basis of our results, etoposide chronic exposure is able to select a population of chemoresistant neuroblastoma cells overexpressing crucial genes of the MDR system and displaying an important GSH-mediated antioxidant defense that efficiently prevents the membrane lipoperoxidative impairment and cell death.

Interestingly, the modulation of GSH levels shows that BSO (an inhibitor of GSH biosynthesis) plus etoposide treatment markedly stimulates H_2_O_2_ over-production and decreases the high tumorigenic potential of HTLA-Chr cells whilst NAC (a donor of cysteine groups) plus etoposide treatment reduces peroxide generation and increases the tumorigenicity. Our data confirms the important role of GSH in the sensitization of neuroblastoma [64–69], ovarian cancer [70], acute lymphoblastic leukemia [71] and other human cancer cells [72, 73] to traditional anticancer therapies.

Since it has been recently reported that the evaluation of oxidative stress status could be a marker of drug efficacy in cancer patients [17], we propose the monitoring of GSH levels and of GSH-related enzymes as potential tools to predict and control the patient's response to therapy and to early identify the onset of drug resistance.

However, further studies are still needed to identify the molecular mechanisms underlying these cell responses which have not yet been fully explained. In fact, in considering that several molecules involved in the response of tumor cells to chemotherapy are redox-modulated, given that they have functional cysteine groups and control key steps of the cell cycle and death, we believe that the identification of these “redox sensors” could be most useful in both the diagnosis and therapy of neuroblastoma.

## MATERIALS AND METHODS

### Cell cultures and treatments

The MYCN-amplified human stage-IV neuroblastoma cell line, HTLA-230, was obtained from Dr. V. Pistoia (G. Gaslini Institute, Genoa, Italy). The cell line was tested for mycoplasma contamination (Mycoplasma Reagent Set, Aurogene s.p.a, Pavia, Italy). After thawing and eight passages in the culture, cell morphology and proliferation were analyzed. Cells were cultured in RPMI 1640 (Euroclone SpA, Pavia, Italy) supplemented with 10% fetal bovine serum (FBS; Euroclone), 2 mM glutamine (Euroclone), 1% penicillin/streptomycin (Euroclone), 1% sodium pyruvate (Sigma), and 1% of aminoacid solution (Sigma).

The Etoposide-Resistant cell line (HTLA-Chr) was selected by treating HTLA-230 cells for 6 months with increasing concentrations of etoposide (Calbiochem, Merck KGaA, Darmstadt, Germany; up to 1.25 μM), and then maintaining them in a medium supplemented with 1.25 μM etoposide, the dose comparable to that clinically used [18]. In parallel, a DMSO-R cell line was selected by treating HTLA-230 cells for 6 months with the concentration of DMSO used to dissolve etoposide.

Parental HTLA-230 and HTLA-Chr cells were treated for 24 hrs with etoposide doses ranging from 1.25 to 100 μM. In other experiments, cells were treated for 24, 48 and 72 hrs with increasing concentrations of doxorubicin (Sigma; 0.046-14.72 μM) or exposed for 3 hrs to increasing concentrations (250-1000 μM) of H_2_O_2_ (Carlo Erba, Milan, Italy). In another series of experiments, parental and HTLA-Chr cells were pre-treated for 1 hr with 1 mM L-Buthionine sulfoximine (BSO, Sigma) or with 2 mM N-Acetylcysteine (NAC, Sigma) and then exposed to etoposide (1.25 and 50 μM) for 24 hrs.

The stock solutions of etoposide were prepared in DMSO and pilot experiments demonstrated that the final DMSO concentrations did not change any cell responses analyzed.

### Cell proliferation assay

Cell proliferation was evaluated by staining cells with carboxyl fluorescein succinimidyl ester (CFDA-SE, Invitrogen, Milan, Italy), a lipophilic dye that reacts with amino groups on peptides and proteins forming a stable amide bond, and detection by dye dilution assay in flow cytometry [68, 74, 75]. Cells were seeded in six-well plates (Corning Incorporated, NY, USA), washed and incubated with 5 μM CFDA-SE in 10 mM PBS in the dark at 37 °C in 5% CO_2_ for 5 min. At the end of incubation, the cells were washed three times with 10 mM PBS supplemented with 1% FBS. Then, the samples were exposed to the treatments. After 24 and 48 hrs, cells were washed and scraped-off in PBS and the intensity of CFDA-SE fluorescence was evaluated by flow cytometry. Proliferation of CFDA-SE-labeled cells was estimated by the progressive halving of cellular fluorescence as every cell division was completed. Samples were analyzed using a FACSCanto II flow cytometer by FacsDiva software version 6.0 (Becton Dickinson Italia, BD, Milan, Italy). The flow cytometry data files were analyzed using the Proliferation Wizard module of the ModFit LT 3.2 software (Verity Software House Inc., Topsham, ME, USA). The proportions of proliferated cells at each division were obtained by ModFit analysis, which generates histograms of fluorescence intensity by applying deconvolution algorithms [76]. The proliferation index is the ratio of the total number of divisions over the number of cells which divided. Each experiment was performed three times.

### Clonogenic assay

Neuroblastoma cells (200 per well) were seeded in 6-well plates (Corning), left to attach as a monolayer and then treated. Subsequently, the medium was changed and the cells were maintained in drug-free medium for 20 days. Cells were then fixed with methanol and stained with crystal violet (0.5 % in water with 50% methanol). Only colonies containing more than 30 cells were considered and the images were acquired with a Nikon Coolpix L22 camera (NIKON Corporation, Tokyo, Japan).

### Immunoblot analysis

Immunoblots were carried out according to standard methods [77] using rabbit antibody anti-PARP (Cell Signalling Technology Inc., Danvers, MA, USA Upstate, Lake Placid, NY, USA), anti-H2AX, anti-GCLC and anti-GCLM (Abcam, Cambridgeshire, UK) and mouse antibody anti-b-actin (Sigma) and anti-γ-H2AX (Abcam).

Anti-mouse and anti-rabbit secondary antibodies were coupled with horseradish peroxidase (GeHealthcare, Buckinghamshire, UK). Proteins were visualized with an enzyme-linked chemiluminescence detection kit according to the manufacturer's (GeHealthcare) instructions. Chemiluminescence was monitored by exposure to film and the signals were analyzed under non-saturating conditions with an image densitometer connected to Quantity One software (Bio-Rad Laboratories, Hercules, CA, USA).

### HPLC evaluation of etoposide levels

Etoposide was measured by HPLC with UV detection. The method was a modification of the method of Haim et al [78]. Aliquots of cell medium and of supernatant of cell extracts were diluted 1:1 with methanol, in order to obtain a 50% methanol solution [79]. HPLC was equipped with a Waters Spherisorb ODS2 column (particle diameter 5μm) [80]. The isocratic elutions were performed with a mobile phase constituted by 0.01M Na acetate buffer pH 3.8 and acetonitrile in 7/3 ratio. The flow rate was 1ml/min and the detection was by absorbance at 250 nm. Peak identification and quantification were made by peak comparison with standard etoposide.

### MTT assay

Cell viability was determined using the dimethylthiazolyl-2-5-diphenyltetrazolium bromide (MTT, Sigma) staining. Briefly, cells were seeded into 96-well plates (Corning) and then treated. Next, the cells were incubated with 0.5 mg/ml MTT for 3 hrs at 37°C. After incubation, the supernatant was discarded, insoluble formazan precipitates were dissolved in HCl (0.1 N in isopropanol) and the absorbance at 570/630 nm was recorded using a microplate reader (EL-808, BIO-TEK Instruments Inc., Winooski, Vermont, USA).

### Detection of hydrogen peroxide (H_2_O_2_) production

After treatment, cells were incubated with 5 μM 2'-7' dichlorofluorescein-diacetate (DCFH-DA; Sigma) and the accumulation of dichlorofluorescein (DCF) was analyzed by flow cytometry using a FACSCanto II flow cytometer (BD) using FlowJo (Tree Star, Inc). At least 10,000 events were analyzed.

### Evaluation of oxygen consumption rate

In order to measure the respiratory activity, 2x10^5^ cells were used for each experiment, using an amperometric O_2_ electrode in a closed chamber, magnetically stirred at 37 °C. The cells were suspended in a medium containing: 137 mM NaCl, 5 mM KH_2_PO_4_, 5 mM KCl, 0.5 mM EDTA, 3 mM MgCl_2_ and 25 mM Tris–HCl, pH 7.4, and permeabilized with 0,3% digitonin for 10 min. Then, the sample was transferred to the chamber and to measure the maximum respiration rate, 5 mM pyruvate plus 2.5 mM malate were added.

### Evaluation of ATP synthesis

ATP synthesis was measured by the highly-sensitive luciferin/luciferase method [81]. Assay was carried out at 37°C over 2 min. by measuring formed ATP from added ADP. Cells were incubated for 10 min. in a medium containing: 10 mM Tris-HCl (pH 7.4), 50 mM KCl, 1 mM EGTA, 2 mM EDTA, 5 mM KH_2_PO_4_, 2 mM MgCl_2_, 0.6 mM Ouabain, 0.040 mg/ml Ampicillin, 0.2 mM di(adenosine-5′) penta-phosphate, 0.2 mM and 5 mM pyruvate plus 2.5 mM malate. Afterwards, ATP synthesis was induced by the addition of 0.3 mM ADP. The ATP content was measured using the luciferin/luciferase ATP bioluminescence assay kit CLSII (Roche, Basel, Switzerland) on a Luminometer (Triathler, Bioscan,Washington, D.C.). ATP standard solutions (Roche, Basel, Switzerland) in the concentration range of 10^−10^-10^−7^ M were used for calibration [82].

### Assay of intracellular levels of ATP and AMP

Cells were washed twice with PBS and lysed with 2.5% perchloric acid (PCA) to block all enzymatic activities. After centrifugation, supernatants containing PCA were collected and neutralized with 0.2 M K_2_CO_3_.

ATP was assayed, following NADP reduction at 340 nm. The medium contained 50 μg of neutralized cell homogenate, 50 mM Tris-HCl pH 8.0, 1 mM NADP, 10 mM MgCl_2_, and 5 mM glucose in 1 ml final volume. Samples were analyzed by spectrophotometer before and after the addition of 4 mg of purified hexokinase/glucose-6-phosphate dehydrogenase.

AMP was evaluated following the NADH oxidation at 340 nm. The medium contained 50 μg of neutralized cell homogenate, 100 mM Tris-HCl (pH 8.0), 75 mM KCl, 5 mM MgCl_2_, 0.2 mM ATP, 0.5 mM phosphoenolpyruvate, 0.2 mM NADH, 10 IU adenylate kinase, 25 IU pyruvate kinase, and 15 IU of lactate dehydrogenase [83].

### Evaluation of extracellular lactate

Lactate concentration was assayed by spectrophotometric analysis in the growth medium, following the reduction of NAD^+^ at 340 nm [84]. The assay medium contained 100 mM Tris/HCl (pH 8), 5 mM NAD^+^ and 1 IU/ml of lactate dehydrogenase. Samples were analyzed before and after the addition of 4 μg of purified lactate dehydrogenase. Data was normalized to the cell number.

### Lactate dehydrogenase activity assay

Lactate dehydrogenase activity (LDH) was measured in the cell homogenate, obtained after sonication in PBS. The assay medium contained 100 mM Tris pH 7.4, 0.15 mM NADH and 10 mM pyruvate. The enzymatic activity was monitored for 5 min., at 340 nm following the oxidation of NADH (angular coefficient for NADH at 340 nm is ε=6.22 mM^−1^ cm^−1^) [85].

### Evaluation of glucose consumption

Glucose consumption was evaluated by measuring supernatant concentration using a double beam spectrophotometer (UNICAM UV2, Analytical S.n.c., PR, Italy), by the hexokinase (HK) and glucose 6 phosphate dehydrogenase (G6PD) coupling system, following the reduction of NADP at 340 nm. The assay medium contained 100 mM Tris HCl, pH 7.4, 2 mM ATP, 10 mM NADP, 2 mM MgCl_2_, 2 IU of HK and 2 IU of G6PD. The reaction was started after the addition of 5 μl of cell medium.

### Gene expression analysis by cDNA microarray

The expression of 18,401 human genes was tested by cDNA microarray. Custom microarrays, made available by the Microarray Department-University of Amsterdam, were used [86, 87]. The whole list of spotted genes is available on the website http://www.micro-array.nl/libraries.html. All data is MIAME-compliant as detailed on the MGED Society website http://www.mged.org/Workgroups/MIAME/miame.html. Used microarrays have been made available by the Microarray Department of the University of Amsterdam (http://www.micro-array.nl).

Purified RNA underwent reverse transcription and amplification using quantitative real-time PCR (qPCR) prior to probe synthesis for array hybridization. A retrotranscription protocol (SuperSMART, Clontech, Palo Alto, CA, USA) was applied as follows: RNA was incubated with a mix of a target sequence-oligo(dT)-linked primer and a target sequence-oligo(dG)-linked at 72°C for 5 min. and then a mastermix solution was added, containing reverse transcriptase and dNTPs mix. Samples were then incubated at 42°C for 90 min. Reaction was terminated by adding ethylenediaminetetraacetic acid, mixture diluted in phosphate buffer and synthesized cDNA purified by column chromatography using a commercially-available purification kit (QIAquick PCR purification kit, Qiagen, Chatsworth, CA, USA).

The amplified cDNA, as purified by column chromatography, was converted into aminomodified oligonucleotides purified by column chromatography and alcohol precipitation and then labelled with fluorescent tracers Cy3 or Cy5 by incubation at room temperature in the dark for 90 min. Fluorescent oligonucleotides were precipitated by cold ethanol and sodium acetate, and then purified by column chromatography. The efficacy of the procedure was checked by spectrophotometric analysis measuring absorbance at 550 (Cy3) and 650 (Cy5) nm. Standardized amounts of labelled probes were hybridized on glass cDNA microarrays. Probes were lyophilized, diluted in 4 μl of EDTA 10 mM and incubated at 95°C for 10 min. Hybridization solution (18 μl) was added and the labelled probe was mixed together to a final volume of 44 μl. The mixture was then transferred onto microarrays which were then covered with a coverslip and hybridized overnight at 50°C in a Hybridization Cassette (Life Technologies, Carlsbad, CA, USA). After 16 hrs, the microarrays were washed twice in a low stringency wash buffer and twice in a high stringency wash buffer. The microarrays were dried in a centrifuge and the signal was acquired by a laser scanner (ScanArray, PerkinElmer, Waltham, MA, USA). Data analysis was performed subtracting the local spot background for each microarray from raw spot intensity, log transformation, normalization per chip and per array (GeneSpringH software, Agilent Technologies, Santa Clara, CA). Each gene was spotted in quadruplicate on the used microarray. Accordingly, results represent the mean among 4 sets of data. Data generated for each mRNA were compared among the various experimental groups by volcano-plot analysis taking into account thresholds of 1.5-fold variation and statistical significance (p 0.05) as evaluated by ANOVA after Bonferroni multiple-testing correction. Global mRNA expression profiles were compared by bidimensional principal component analysis of variance (PCA).

### Real time RT PCR

SYBR green real time RT-PCR was performed for the validation of the data produced by microarray analysis. The primer sequences (TIB Molbiol, Italy) used are the following: ARNT (F 5′-GATGCGATGATGACCAG ATGTG-3′; R 5′-CAGTGAGGAAAGATGGCTTGTAGG-3′); EZRIN (F 5′-CGCAAGGAGGATGAAGTT-3′; R 5′-GGATGATGTCATTGTGGGTC-3′), ABC B6 (F 5′-CAGCAGGGACAGGAAGAA-3′; R 5′-CCAAGACCAGGATGAAAT-3′) and BCRP-1 (F 5′-TGGCTGTCATGGCTTCAGTA-3′; R 5′-GCCACGTGATTCTTCCACAA-3′). To minimize primer-dimer formation, primer set concentration and thermocycling conditions were optimized (data not shown). cDNA was generated by using the Superscript II Reverse Transcription (LifeTechnologies, Carlsbad, California, USA) and following the manufacturer's instructions. Then, the reaction mixture was incubated at 65°C for 5 min. and then transferred into ice, centrifuged and added with 8 μl of the buffer containing 5x first strand buffer, 0.1M DTT, RNAse OUT and Superscript II.

The samples were incubated for 60 min. at 42°C and then, for 15 min. at 70°C. PCRs were performed on a Rotor-Gene 3000^§^ (Corbett Research, 1/14 Hilly St, Mortlake NSW 2137, Australia). Each reaction was carried out in a mixture containing 10x PCR buffer, 50mM MgCl_2_, dNTM mix, 10 μM primer A, 10 μM primer S, Platinum^®^ Taq DNA polymerase (LifeTechnologies), RT (cDNA 1:10 diluted) and 3x SYBR GREEN^®^ (LifeTechnologies). The thermal profile consisted of hot-start enzyme activation at 95°C for 2 min., followed by 45 cycles of PCR which consisted of the denaturation at 94°C for 45s, the annealing for 30s (temperature reaction depends on the gene), and the elongation at 72°C for 30s. Each strain used was tested in triplicate. Each sample was submitted to two distinct reactions of q-PCR, with a pair of primers flanking the sequence of the gene of interest and the other with the pair of primers of house-keeper gene in order to normalize the relative amount of the gene of interest.

### Catalase and SOD activity analysis

The enzymatic activity of catalase and SOD was evaluated by Catalase Activity Colorimetric/Fluorimetric Assay kit (BioVision, BioVision Incorporated, CA, U.S.A) and Superoxide Dismutase Activity Assay kit (BioVision), respectively, following the manufacturer's instructions. Briefly, after treatments, 10^6^ cells were homogenized in assay buffer (for catalase activity) or in ice-cold 0.1 M Tris/HCl, pH 7.4 containing 0.5% Triton X-100, 5 mM Beta-mercapto-Ethanol and 0.1 mg/ml PMSF. Subsequently, cell lysates were centrifuged and the supernatants were collected and used to evaluate the enzyme activities.

### Reduced (GSH) and oxidized (GSSG) glutathione levels

After treatments, cells were detached and harvested in PBS solution and about 25% of cell suspension was used for protein dosage, the remaining 75% of cell suspension was mixed with the same volume of the precipitating solution (2 mM EDTA, 0.61 N TCA and 0.02 N HCl) and centrifuged (Euroclone) at 4,000 rpm for 15 min. at 4°C.

The resulting supernatant was used to evaluate the GSH and GSSG contents by the reaction with o-phthalaldehyde (OPA) [88]. Standard solutions were prepared by dissolving GSH or GSSG in Redox Quenching Buffer (RQB) containing 20 mM HCl, 5 mM DTPA and 10 mM ascorbic acid. Samples or standards (GSH or GSSG) were treated with 5% TCA-RQB solution. To evaluate GSSG levels, N-ethylmaleimide (7.5 mM in RQB) was added. Subsequently, 1 M potassium phosphate (KPi) buffer (pH 7.0) was added to all tubes and the samples were incubated for 5 min. at room temperature. For GSSG assay, 100 mM dithionite-RQB was used and the samples were incubated for 60 min. at room temperature. Then, 0.1 M KPi buffer (pH 6.9) and OPA (5 mg/ml in methanol) were added and, after a 30 min. incubation at room temperature, sample fluorescence was monitored with a Perkin Elmer fluorimeter (Perkin Elmer Life and Analytical Sciences, Shelton, USA) at 365/430 nm.

### RT-PCR analysis

Total RNA was extracted using TRIZOL reagent (LifeTechnologies, Carlsbad, California, USA) according to the manufacturer's instructions. Total RNA (1 μg) was reverse-transcribed into cDNA by a random hexamer primer and SuperScript™ II Reverse Transcriptase (LifeTechnologies).

Amplification of cDNA by a polymerase chain reaction was performed using AmpliTaq Polymerase (LifeTechnologies) and specific primers for *GCLC* F 5′-ATG GAG GTG CAA TTA ACA GAC-3′; *GCLC* R 5′-ACT GCA TTG CCA CCT TTG CA-3′ (206 bp); *GCLM* F 5′-CCA GAT GTC TTG GAA TGC-3′; *GCLM* R 5′-TGC AGT CAA ATC TGG TGG-3′(408 bp); *GAPDH* F 5′-AGC CAC ATC GCT CAG ACA CC-3′; and *GAPDH* R 5′-TGA GGC TGT TGT CAT ACT TCT C-3′ (426 bp). Target cDNA was amplified as follows: 5 min. at 95°C and then 30 cycles of amplification (GCLC: denaturation at 95°C for 45s, annealing at 56°C for 45s and extension at 72°C for 45s; GCLM: denaturation at 95°C for 45s, annealing at 54°C for 45s and extension at 72°C for 45s; GAPDH, denaturation at 95°C for 1 min., annealing at 59°C for 1 min. and extension at 72°C for 1 min.). PCR products were separated by electrophoresis on 2 % agarose gel, pre-stained with ethidium bromide, and then visualized under UV light and quantified by densitometric analysis by using a specific software (GelDoc, BioRad, Milan, Italy).

### Glutathione-S-transferase (GST) activity analysis

GST activity was tested according to the method of De Flora et al. [89]. Briefly, cell homogenates, containing 800 μg proteins, were incubated for 40 min. at 37°C using 1-cloro 2,4-dinitroclorobenzene and reduced GSH (Sigma) as substrates. This reaction, catalyzed by GST, resulted in the production of conjugated 5-thio-2-nitrobenzoic acid whose absorbance was evaluated at 340 nm using a nanospectrophotometer (Nanodrop ND-1000, Nanodrop technologies, Inc. Wilmington, DE USA).

### Malondialdehyde assay

Malondialdehyde (MDA) levels were analyzed by using thiobarbituric acid reactive substance (TBARS) assay with minor modifications [90]. This method is based on the reaction of MDA, a breakdown product of lipid peroxides, with TBA. To evaluate the basal concentration of MDA, 50 μg of total protein dissolved in 300 μl of milliQ water were added to 600 μl of TBARS solution containing 15% TCA in 0.25 N HCl and 26 mM TBA. The mixture was incubated for 40 min. at 100 °C, centrifuged at 14,000 rpm for 2 min., and the supernatant was analyzed by spectrophotometer at 532 nm. Different MDA concentrations (0.75, 1, and 2 μM) were used to obtain a standard curve.

### Data analysis

Results were expressed as mean ± SEM from at least three independent experiments. The statistical significance of parametric differences among the sets of experimental data was evaluated by one-way ANOVA and Dunnett's test for multiple comparisons.

## SUPPLEMENTARY FIGURES


