# Supplementary material for: Glutathione-mediated antioxidant response and aerobic metabolism: two crucial factors involved in determining the multi-drug resistance of high-risk neuroblastoma
Source: Oncotarget. 2016 Sep 23;7(43):70715–37. doi: 10.18632/oncotarget.12209 (PMC5342585; doi:10.18632/oncotarget.12209)
Supplement: Supplementary file 1 [file oncotarget-07-70715-s001.pdf]

Glutathione-mediated antioxidant response and aerobic metabolism: two crucial factors involved in determining the multi-drug resistance of high-risk neuroblastoma

SUPPLEMENTARY FIGURES

A)

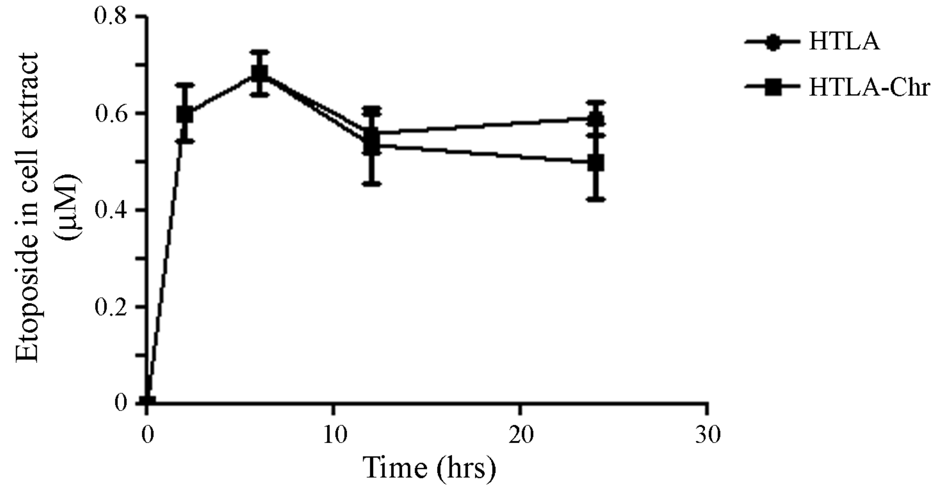

B)

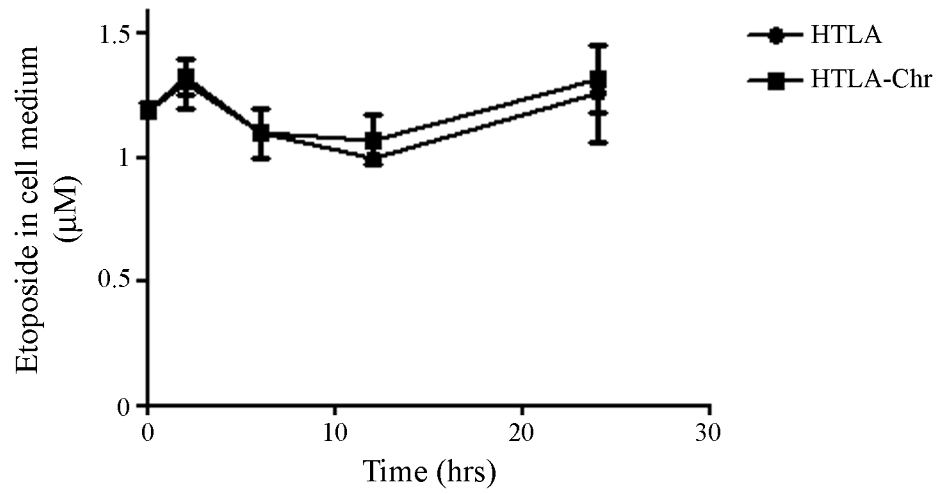

Supplementary Figure S1: HTLA parental and HTLA-Chr cells are able to internalize the same amounts of etoposide. **A.** Etoposide levels were evaluated by HPLC analysis in the cell extracts of HTLA and HTLA-Chr cells treated for 2, 6, 12 and 24 hrs with the drug (1.25 µM). The graph summarizes quantitative data of the means ± S.E.M. of three independent experiments. **B.** Etoposide levels were evaluated by HPLC analysis in the medium of HTLA and HTLA-Chr cells treated for 2, 6, 12 and 24 hrs with the drug (1.25 µM). The graph summarizes quantitative data of the means ± S.E.M. of three independent experiments.

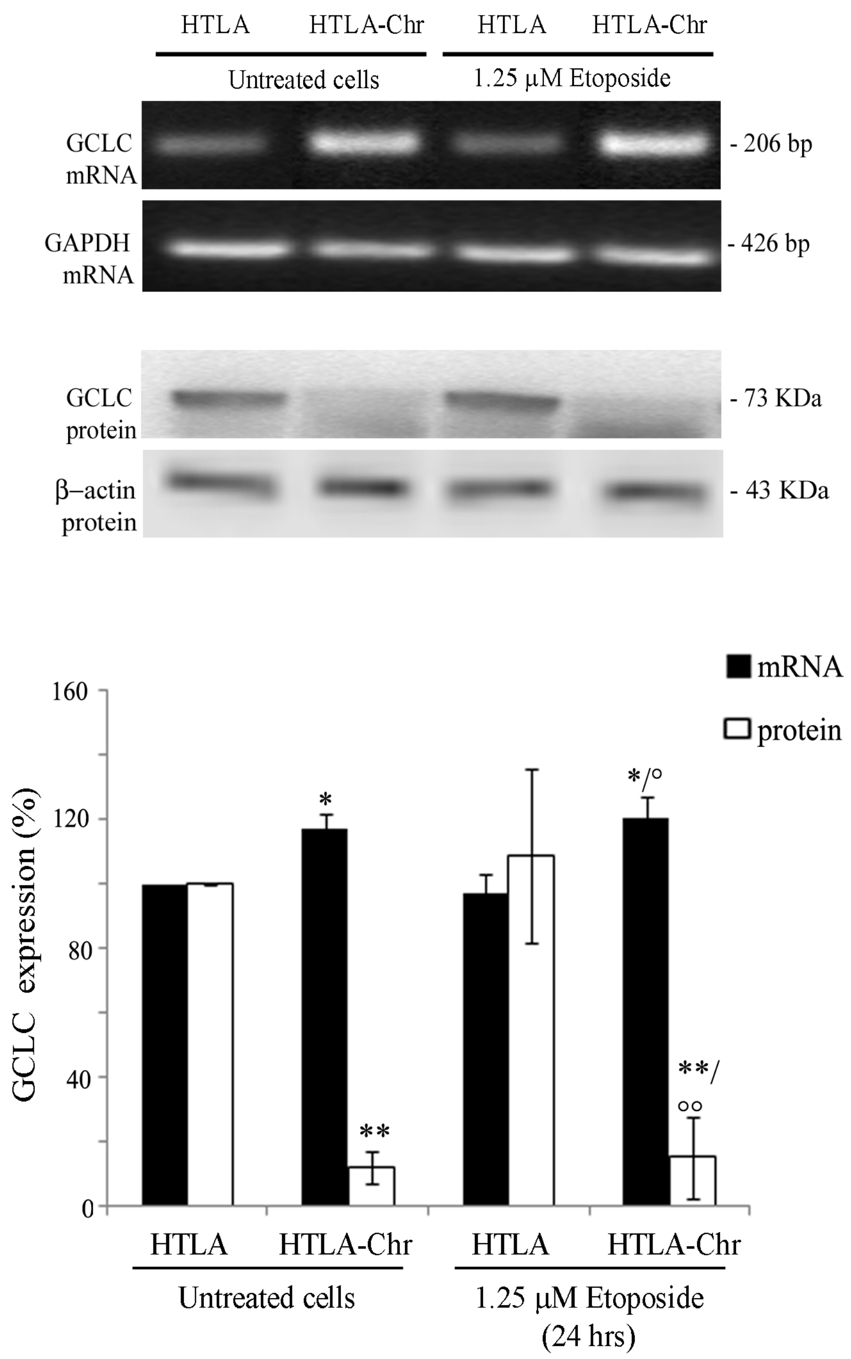

Supplementary Figure S2: HTLA-Chr cells have an high level of GCLC mRNA expression and a marked reduction of GCLC protein in respect to the parental cells. RT-PCR (upper panels) and immunoblot (lower panels) analyses of the catalytic subunit of γ-glutamyl-cysteinyl ligase (GCLC) in untreated and in 1.25 μM etoposide-treated HTLA and HTLA-Chr cells. Glyceraldehyde 3-phosphate dehydrogenase (GAPDH) signal and β-actin expression are the internal loading controls for mRNA and protein, respectively. The images are representative of three independent experiments with essentially similar results. Histograms summarize quantitative data of means, normalized to GAPDH/β-actin expression ± S.E.M. of three independent experiments. **p*<0.05 vs. untreated HTLA cells; ***p*<0.01 vs. untreated HTLA cells; °*p*<0.05 vs. 1.25 μM etoposide-treated HTLA cells; °°*p*<0.01 vs. 1.25 μM etoposide-treated HTLA cells.
